# Aptamer loaded superparamagnetic beads for selective capturing and gentle release of activated protein C

**DOI:** 10.1038/s41598-022-11198-5

**Published:** 2022-04-30

**Authors:** Nasim Shahidi Hamedani, Felix Lucian Happich, Eva-Maria Klein, Heiko Rühl, Günter Mayer, Johannes Oldenburg, Jens Müller, Bernd Pötzsch

**Affiliations:** 1 Institute of Experimental Hematology and Transfusion Medicine, University Hospital Bonn, 53127 Bonn, Germany; 2 Life and Medical Sciences Institute, University of Bonn, 53115 Bonn, Germany

**Keywords:** Biological techniques, Isolation, separation and purification

## Abstract

Activated protein C (APC) is a serine protease with anticoagulant and cytoprotective activities which make it an attractive target for diagnostic and therapeutic applications. In this work, we present one-step activation of APC from a commercial source of protein C (PC, Ceprotin) followed by rapid and efficient purification using an APC-specific aptamer, HS02-52G, loaded on MyOne superparamagnetic beads. Due to the Ca^2+^-dependent binding of APC to HS02-52G, an efficient capturing of APC was applied in the presence of Ca^2+^ ions, while a gentle release of captured APC was achieved in the elution buffer containing low EDTA concentration (5 mM). The captured and eluted APC showed more than 95% purity according to SDS-PAGE gel analysis and an enzyme-linked fluorescent assay (VIDAS Protein C). The purification yield of 45% was calculated when 4.2 µg APC was used, however this yield reduced to 21% if the starting amount of APC increased to 28.5 µg. Altogether, this method is recommended for rapid and efficient PC activation and APC purification. The purified APC can be used directly for downstream processes where high concentration of pure and active APC is needed.

## Introduction

Human protein C (PC) is a two-chain protein which circulates in plasma predominantly as a zymogen with the molecular weight of 56.2 kDa. Activated protein C (APC) is an anticoagulant serine protease derived from PC after proteolytic activation by thrombin^1^. In physiological conditions the plasma concentration of PC is 70 nM, while the concentration of its activated form, APC, is estimated as low as 40 pM^2^. Although APC is stable in purified systems for few hours, its catalytic half-life in circulatory blood was estimated as ~ 20 min^3^. APC does not only have antithrombotic activity, but is also involved in several anti-inflammatory and cytoprotective processes to maintain the health and integrity of the vasculature^4^. This area of functionality makes APC an attractive target for comprehensive studies of diagnostic applications along with therapeutic approaches targeting multiple indications of APC in both molecular and cellular states.

Access to a reliable and perpetual source of purified proteins for downstream procedures is a main concern in molecular biology laboratories^5,6^. Either recombinant production of a protein or isolation from biological fluids such as plasma requires a crucial step of protein purification. Proteins are purified on the basis of characteristics such as solubility, molecular weight, charge, and/or specific binding affinity. Usually, protein mixtures are subjected to sequential series of separations, each based on a different property of protein to yield a high ultimate purity^5^. A variety of purification techniques are available such as salting out, dialysis, electrophoresis, size-exclusion chromatography, ion-exchange chromatography, affinity chromatography, and high pressure liquid chromatography^7–9^. These techniques might result in purified proteins. However, setting successive steps of purification, depends on the initial amount of materials, may yield a very low quantity of a protein and low enzyme activity when an active enzyme is subjected to purification.

Classical purification procedures for PC have been summarized by Kisiel and Davie including two key steps of dextran sulfate-agarose and preparative electrophoresis^10^. During the ensuing years, the major advances with respect to PC isolation from plasma have involved improvements in affinity chromatography by the use of monoclonal antibodies^11^. The common procedure of achieving APC is first PC isolation using affinity chromatography followed by APC generation and purification using size-exclusion chromatography. Similar approaches with minor modifications were applied by Iserman et al. in which, PC was initially isolated from prothrombin complex concentrate (PCC) using antibody-based affinity chromatography. In the following step, the recovered PC was activated using human thrombin and the thrombin added to the activation mixture was separated subsequently using ion-exchange chromatography^12,13^. In another approach, PC was activated by bovine thrombin immobilized on Sepharose 4B beads^14^, however in all above mentioned approaches, the yield of separation and the specific activity of APC remain unknown. In addition, regarding the similar molecular weight and isoelectric point of PC and APC, the risk of co-purification of residual amount of PC must be considered.

Aptamers are single-stranded synthetic oligonucleotides generated by an in vitro selection process termed systematic evolution of ligands by exponential enrichment (SELEX)^15,16^. Aptamers bind to their target molecules with high affinity and specificity and involve the advantage of discrimination between active and inactive structural conformation of an enzyme^17,18^. In the context of affinity separation, aptamers offer advantages over antibodies which suit them for designing aptamer-based affinity purification strategies. While generation and application of antibodies are restricted to physiological conditions, aptamers can be generated under varying conditions during their in vitro selection. This enables the design of affinity ligands that are functional under desired conditions and thus allows adopting aptamers to address the purification problem where affinity ligands might be used under non-physiological conditions^19,20^.

In comparison to tag-specific capture probes (such as Histidine or Glutathione), aptamers are used for the purification of proteins free of genetic modifications. The strength of using aptamers in column affinity chromatography has been shown in various successful applications. In a study by Liu and colleagues, using immobilized aptamers on Sepharose 4B beads, aflatoxin B2 was isolated from peanut samples with high reproducibility and accuracy^21^. In another study a specific aptamer against ochratoxin A (OTA) was immobilized both covalently and non-covalently on Sepharose and agarose beads, respectively. These two approaches showed comparable results to conventional immunoaffinity columns using OTA-specific antibodies to determine OTA levels in red wine samples. Surprisingly, fewer co-isolation of contaminants was observed by using oligo-affinity columns^22^.

Aptamers were used previously in affinity chromatography approaches for purification of various coagulation factors such as Factor VII (FVII), Factor IX (FIX), and complement factor H^19^. In all three approaches the aptamer-loaded Sepharose beads were used for affinity chromatography. The EDTA-induced elution of captured enzymes resulted in extraction of each protein with the purity more than 95%. In addition, FIX which was purified using aptamer showed higher purity in comparison to immunopurified FIX^19^.

Column-based affinity purification strategies currently represent a powerful tool for downstream processing both in terms of selectivity and recovery. However, coping with multi-step purification, in which suspended solid and fouling components are present in the sample, is a major disadvantage of all standard column liquid chromatography procedures. Though, in these cases magnetic affinity techniques have shown their usefulness. Magnetic carriers bearing an immobilized affinity ligand have the advantage of one step capturing of the target protein and rapid and easy elution of the protein after removal of contaminants^23^. Lönne et al. established a purification platform in small scale where V7t1 aptamer immobilized on polystyrene magnetic beads was used for purification of vascular endothelial growth factor^24^. In another study, metal ions such as Ni(II), Cu(II), Co(II), and Zn(II) were immobilized on magnetic poly(hydroxyethylmethacrylate-N-methacryloyl-(L)-histidine nanoparticles which were used for single step isolation and purification of PC from aqueous solutions^25^.

**Figure 1:**
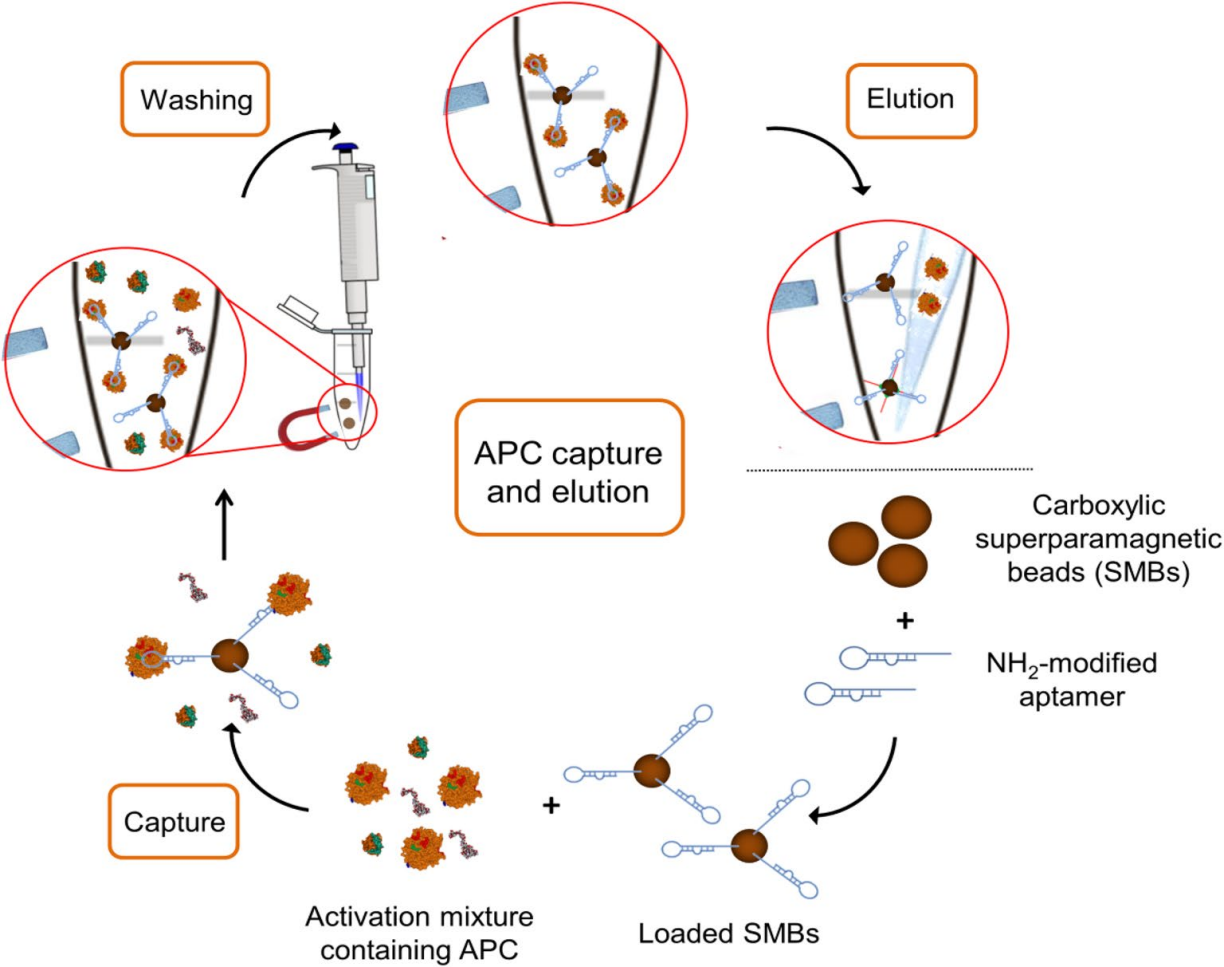
Principle of two-step purification method for capturing and elution of activated protein C (APC) using aptamer-loaded superparamagnetic beads.

In this work the APC-binding DNA aptamer HS02-52G^26^ was covalently coupled to Dynabeads MyOne carboxylic acid superparamagnetic beads (SMBs), generating a functionalized affinity matrix for capturing and purification of APC. The aptamer is directed against the heparin-binding site of APC and showed reduced affinity to PC and human thrombin^18,26^. Oriented immobilization of the aptamer via 3’-terminal amino groups was chosen to maintain the molecular flexibility and three-dimensional aptamer folding. The functionalized magnetic beads were utilized for protein capturing and elution from the activation reagent containing PC, human albumin, human thrombin, and generated APC. The high potential of superparamagnetic beads loaded with APC-specific aptamers allows for the selective capturing and high yield purification of APC (Fig. 1).

## Results and discussion

In order to reduce discrepancies in PC content and to minimize batch to batch variations, Ceprotin which was reconstituted in a NaCl solution provided by the manufacturer at a final concentration of 250 IU/ml was chosen as the starting solution. The concentration of PC in the used batch of Ceprotin was determined by measurement of PC activity (Berichrom Protein C Kit; Siemens Healthcare Diagnostics, Marburg, Germany) and PC antigen (VIDAS Protein C Kit; Biomerieux, Nürtingen, Germany) levels in parallel with characterized PC preparations [HTI], whereat one unit of Ceprotin corresponded to 3.675 µg PC.

Thrombin is the most physiologically relevant activator of PC. In vivo, thrombin binds to its receptor thrombomodulin (TM), while PC binds to endothelial PC receptor (EPCR) which in turn orients and localizes PC to the endothelium. Localization of the thrombin-TM complex to the adjacent proximity of PC-EPCR complex accelerates activation of PC ~ 2000-fold in a Ca^2+^ dependent manner compared to incubation of thrombin and PC in free solution^27–29^. Ca^2+^ ions are required for PC activation in vivo, whereas in the absence of TM, binding of Ca^2+^ to the 70–80 loop of PC inhibits PC activation by thrombin^30^. Accordingly, to avoid the inhibitory effect of Ca^2+^ in thrombin-mediated PC activation in the purified system, a PC activation buffer containing 1 mM EDTA was used. Following further optimization in the PC activation process, for 1 µM (62.5 µg/ml) PC, the optimal thrombin concentration was 85 nM or 3.12 µg/ml (8.5% molar ratio or 5% w/w ratio to PC), whereas higher thrombin concentrations did not result in higher APC generation (see Supplementary Fig. S1a online). This data correlates well with previous work focusing on PC activation and purification from prothrombin complex concentrate (PCC) in which 5% w/w human thrombin to PC was incubated at 37 °C for 3 h to achieve optimum APC generation^13^.

As the next step, the optimum incubation time of thrombin and PC, in which the balance between PC activation and APC degradation is in favor of PC activation, was determined. According to the Supplementary Fig. S1b, APC generation was increased within the first hour of incubation followed by a plateau of high and constant activity of APC up to 24 h. However, the stable APC activity might have resulted either from comparable activation and inactivation rates of APC or from complete activation and negligible inactivation of APC in the absence of its inhibitors.

To evaluate APC stability in the activation buffer at different time points, APC was incubated in the activation buffer up to 5 h and the APC activity was measured in the sub-samples. The data shown in Supplementary Fig. S1c confirmed the stability of APC in activation buffer up to 5 h. Overall, an activation time of 3 h was set for further experiments, however in special occasions which fast APC production is in favor, reduction of the incubation time to 1 h might result in the same APC production.

Although the peptide substrate PCa-5791 is rather specific for APC, this substrate is hydrolyzed in the presence of low nanomolar concentration of thrombin which might conceal the detection of APC generation in the activation mixture (see Supplementary Fig. S2 online). To use PCa-5791 in the activity assay for monitoring of the APC generation, it is important to assure that the substrate is converted only by APC in the activation mixture. Therefore, to prohibit thrombin-mediated PCa-5791 substrate conversion, thrombin was inhibited by the addition of hirudin to the activation mixture after 3 h of PC activation, or to the substrate buffer^31^. The optimum hirudin concentration was determined by incubation of 2.5 µg/ml, 12.5 µg/ml, and 20 µg/ml thrombin (corresponding to 68 nM, 341 nM, and 545 nM thrombin, respectively) with increasing concentrations of hirudin. The IC_50_ values for the used thrombin concentrations were calculated as 0.17 µM, 1.96 µM, and 2.26 µM hirudin, respectively. In total, the hirudin concentration was set as 5-time higher molar ratio in comparison to thrombin to assure the inhibition of thrombin at the end of the activation process (see Supplementary Fig. S1d online).

In order to separate APC from the other ingredients of the PC activation mixture such as thrombin, hirudin, human albumin, and residual PC, aptamer-based affinity separation was used. To achieve this goal, five previously selected and characterized APC-specific aptamers (HS02-52G, NB1-83, NB1-46, NB2-81, and NB2-57G), were chosen.

All of these aptamers showed binding affinity to APC in the low nanomolar range and high specificity towards APC whereas zymogenic PC and thrombin were not captured using these aptamers^18,26^. To identify the best aptamer for APC capturing and elution, Biolayer Interferometry (BLI) assay was performed by loading the streptavidin-coated (SAX) biosensors with each biotinylated aptamer, followed by APC capturing and dissociation in the binding buffer. The data showed that among all aptamers tested, HS02-52G appeared to be the best capturing aptamer as the highest capturing signal was achieved using this aptamer (see Supplementary Fig. S3 and Table S1 online).

The binding forces mediating the aptamer-target interactions are the electrostatic interactions such as hydrogen bonding, electrostatic bonding, the hydrophobic effect, π-π stacking, and van der Waals forces^32^. In addition, it was previously demonstrated that HS02-52G aptamer binds to APC in a Ca^2+^-dependent manner^26^. Therefore, EDTA which was present in the final concentration of 1 mM in activation mixture might interfere with APC capturing by HS02-52G. To avoid this, a buffer-exchange step was added between PC activation and APC capturing steps to remove EDTA.

High salt concentration might either interfere with intermolecular electrostatic interactions between aptamer and target protein or cause structural disruption of aptamer resulting in destabilization of aptamer-target protein bond^33^. Hence, high NaCl concentration was used previously in elution buffers in which detachment of the protein from its specific ligand is required^24,34^. In addition, it was previously demonstrated that HS02-52G aptamer binds to APC in a Ca^2+^-dependent manner^26^ therefore, aptamer-mediated captured APC may be eluted using chelating agent, EDTA. To assess the efficiency of different elution strategies, the BLI method was used in which APC was captured by HS02-52G aptamer immobilized on the SAX-biosensors and the dissociation step was performed in the binding buffer containing either 1 M NaCl or 5 mM EDTA.

**Figure 2:**
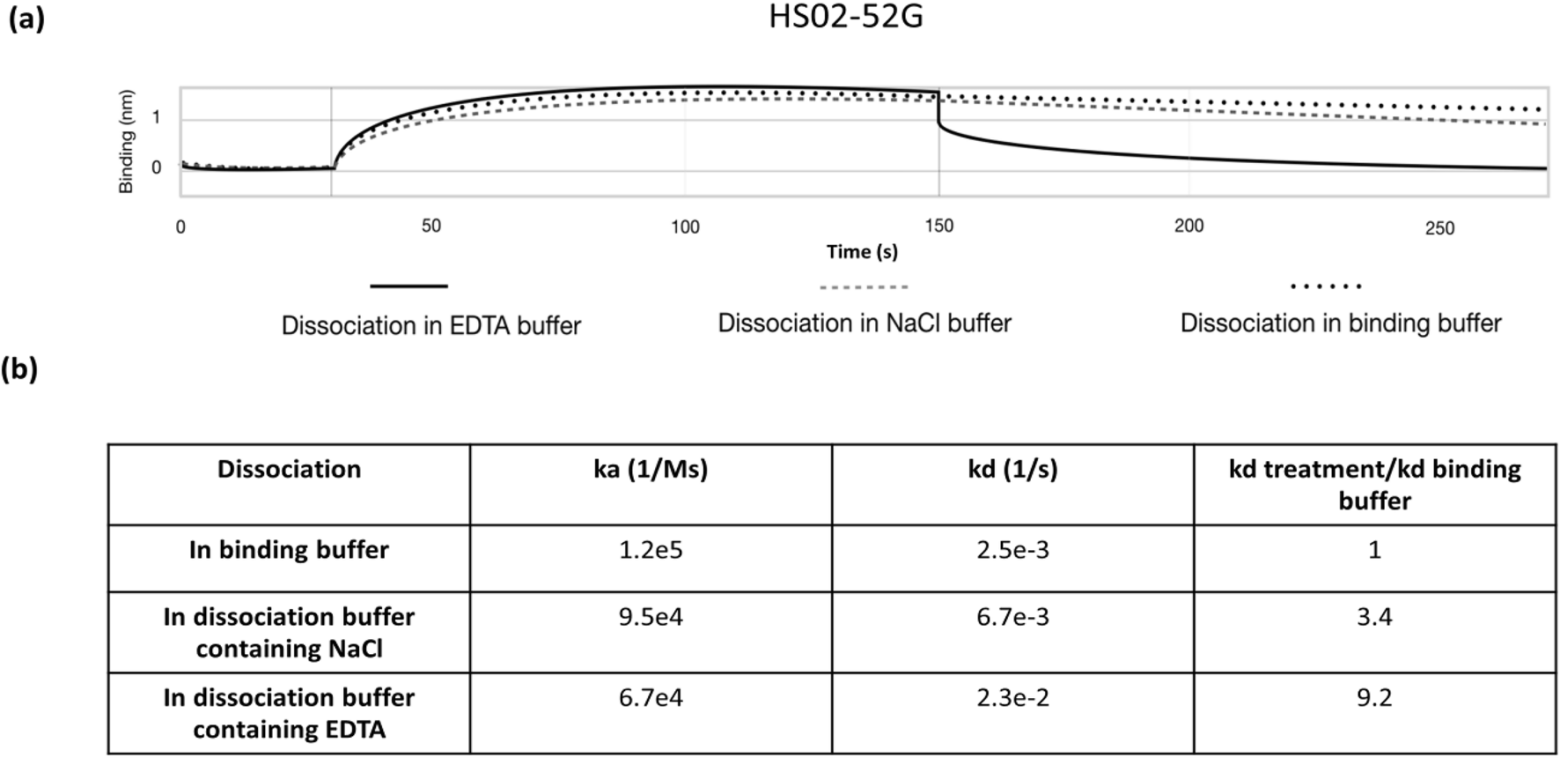
APC association and dissociation from biotinylate-HS02-52G aptamer immobilized on streptavidin-coated biosensors. (**a**) HS02-52G aptamer was immobilized on streptavidin coated biosensors followed by lowering the loaded biosensor first in binding buffer containing 500 nM APC and then in dissociation buffer containing either 5 mM EDTA or 1 M NaCl. (**b**) The association (ka) and dissociation (kd) constants were calculated by local analysis. 0 –30 s, baseline; 30–150 s, association step; 150–270 s, dissociation step.

The obtained data showed that although the calculated association constant was comparable in all three experiments, the dissociation constant (kd) was 9 times higher when the elution buffer 2 containing EDTA was used as dissociation buffer compared to the experiment in which binding buffer was used as elution buffer. This ratio was only 3.4 times higher when elution buffer 1 containing 1 M NaCl was used (Fig. 2). Hence the elution buffer was prepared as 10 mM Tris–HCl, 144 mM NaCl, pH 7.4 containing 5 mM EDTA. This method is applicable only because of the inherent characteristic of HS02-52G and APC binding which is Ca^2+^ dependent and is diminished in the presence of high concentration of chelating agents.

A folded aptamer has a relatively compact structure in which the 3’ or 5’ end might be embedded inside the tertiary structure. In addition, chemical grafting to the 3’ or 5’ end can alter the tertiary structure of the aptamer which might be necessary for target binding. It is therefore essential to introduce a spacer arm between the aptamer sequence and the grafted functional group to reduce the structural changes and to facilitate the coupling to the sorbent. We previously showed that grafting of small molecules such as biotin with a spacer arm (tetraethylene glycol, TEG) did not interfere with the binding affinity and specificity of the aptamers to APC as the 3’-end biotinylated aptamers, which were immobilized on the streptavidin-coated biosensors for the BLI assay (see Supplementary Fig. S3 online) or coated in the maxisorb plates, easily captured their target molecule, APC^18,26^. In addition, these studies showed that the functional groups such as biotin grafted to the 3’ end of aptamer sequence retain their functionality, demonstrating that the 3’ end of the aptamer is accessible after tertiary structure formation^26,35,36^. Hence, in the present study, the HS02-52G aptamer was modified on the 3’-end by attachment of first a hexaethylene glycol (HEG) spacer and then a primary amine (-NH_2_) functional group.

Due to predicaments about how to select the most appropriate grafting to immobilized aptamer on the solid-phase carrier, loading of biotinylated oligonucleotides on streptavidin-coated SMBs was investigated previously in our lab^35^. This study showed that although the biotin-streptavidin binding is the strongest non-covalent bond in nature, a femtomole amount of streptavidin was released from the SMB during the first elution step^35^. This not only introduces a source of protein contamination to the downstream processes, but also reduces the efficiency of capturing by successive use of the loaded beads. This problem can be easily removed by grafting aptamer molecules to the solid carrier using covalent binding^37^. In this respect, an amide-bond formation strategy was used by grafting of NH_2_-modified aptamer to the COOH-coated magnetic beads.

According to the fact that the oligonucleotide structure does not contain a carboxylic acid group, the one-step protocol for activation and loading of primary amine-modified aptamers on magnetic beads containing carboxylic acid groups on the surface was used. Five hundred microlitres of Dynabeads MyOne Carboxylic Acid beads (MyOne) containing 5 mg beads or 500 µl of Dynabeads M-270 Carboxylic Acid beads (M-270) containing 15 mg beads were mixed with 25 nmol amino-modified HS02-52G aptamer in the presence of 500 mM EDC and incubated overnight. The residual functional groups on the surface of magnetic beads were blocked using 250 mM Tris–HCl blocking buffer. The coupling of aptamer molecules on SMBs was confirmed by flow cytometry experiement in which loaded SMBs were first incubated with SYBR Green (1:10,000 dilution) and then identified by corresponding FCS/SSC- or SYBR Green-positive events (FL1) using a Navios EX flow cytometer (Beckman Coulter, Krefeld, Germany). The data showed in Supplementary Fig. S4 confirmed coupling of HS02-52G or AD02-52 aptamer molecules to the MyOne beads. The coupled beads showed a peak shift after incubation with SYBR Green while non-coupled beads showed overlapping peaks bearing minimum peak displacement.

MyOne beads was provided in a suspension of 10 mg beads/ml, bead density of 7-12e9 beads/ml and bead diameter of 1 µm; therefore, 5 mg of MyOne beads offers ~ 142 cm^2^ surface for aptamer binding. On the other hand, M-270 beads was delivered in suspension form having 30 mg beads/ml, bead density of 2e9 beads/ml and bead diameter of 2.8 µM; hence 15 mg of M-270 beads offer 246 cm^2^ interacting surface due to their longer diameter and larger spherical shape. Coupling of NH_2_-modified HS02-52G (25 nmol) on MyOne beads resulted in 66.4 µg (4.1 nmol, 16.4% of starting aptamer amount, 820 pmol/mg bead) aptamer immobilization on the beads corresponding to 13.3 µg aptamer/mg of the beads. On the other hand, trying to immobilize the same amount of aptamer on M-270 beads resulted in 54 µg (3.34 nmol, 13.4% of starting aptamer amount, 223 pmol/mg bead) aptamer immobilization on the beads corresponding to 5.38 µg aptamer/mg of the beads (see Supplementary Table S2 online). This means that, although the M-270 beads offer a larger interactive surface, the yield of aptamer immobilization on their surface is rather lower than MyOne beads. This may show that smaller spherical particles with higher density offer a more accessible interactive surface with less steric hindrance for aptamer coupling.

A low yield of coupling (≤ 20%) was also described previously by Liu et al., where the coupling ratio of aptamer immobilization on CNBr-activated Sepharose beads was only 17%^21^. In addition, Lim et al. described the efficiency of immobilization of His-tag-specific aptamer on aminomethylated polystyrene resin as 510 fmol/mg bead^38^. One possible explanation is that the acidic character of aptamers, due to the ionizable phosphate groups and their negative net charge, likely produce charges repulsion between the aptamer and COOH-modified solid sorbent. This electrostatic repulsion might slow down the aptamer-bead grafting reaction and affect the grafting yield negatively^39^. However, by loading of 6H7 aptamer on BioMag Carboxyl-terminated magnetic beads, Zhu et al. reported an increased grafting efficiency of 157 pmol/mg to 1037 pmol/mg when aptamer concentrations increased from 10 µM to 40 µM, respectively. The loading efficiency was superior to the yield we achieved, but it did not necessarily reflect the yield of capturing of His-tag protein because a 1:1 protein:aptamer molar ratio of capturing was achieved only by using the beads with the lowest surface aptamer density (157 pmol/mg beads) and the protein:aptamer ratio was reduced to 1:5 when using beads with higher aptamer density of 1037 pmol/mg^34^.

**Figure 3:**
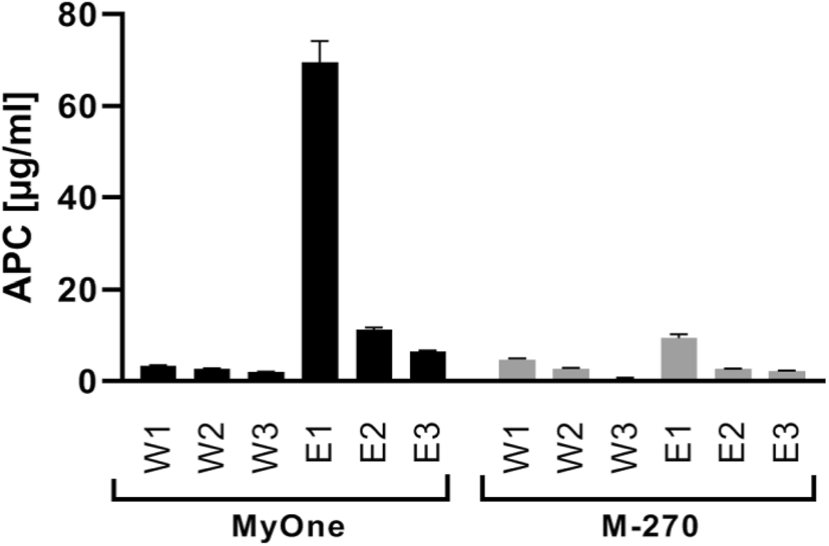
APC capturing and elution using two different aptamer-loaded superparamagnetic beads. HS02-52G aptamers were coupled to Dynabeads MyOne Carboxylic Acid beads (MyOne) or Dynabeads M-270 Carboxylic Acid beads (M-270) followed by incubation of the beads with a binding buffer containing 10 µg APC in 500 µl of binding buffer (5 µg/ml). The APC content in consecutive steps of washing (W1 to W3) and elution (E1 to E3) was quantified using the APC activity assay and an APC standard curve. Data are shown as mean ± SD of three measurements.

The capturing capacity of aptamer-loaded magnetic beads is not only dominated by the aptamer density coupled on the surface of magnetic beads but also by the steric hindrance among neighbored aptamers; thus, the capturing capacity varied from lower amount to maximum 1:1 molar ratio of ligand:target. Therefore, the efficiency of capturing and elution of 10 µg APC was determined using both MyOne and M-270 beads. MyOne beads showed higher efficiency of capturing as in the first elution step a higher amount of APC was released indicating more efficient capturing capacity (70 µg/ml APC in E1 using MyOne compared to 9.5 µg/ml APC concentration in E1 by using M-270 beads) (Fig. 3). Hence, MyOne beads were chosen for further characterization.

**Figure 4:**
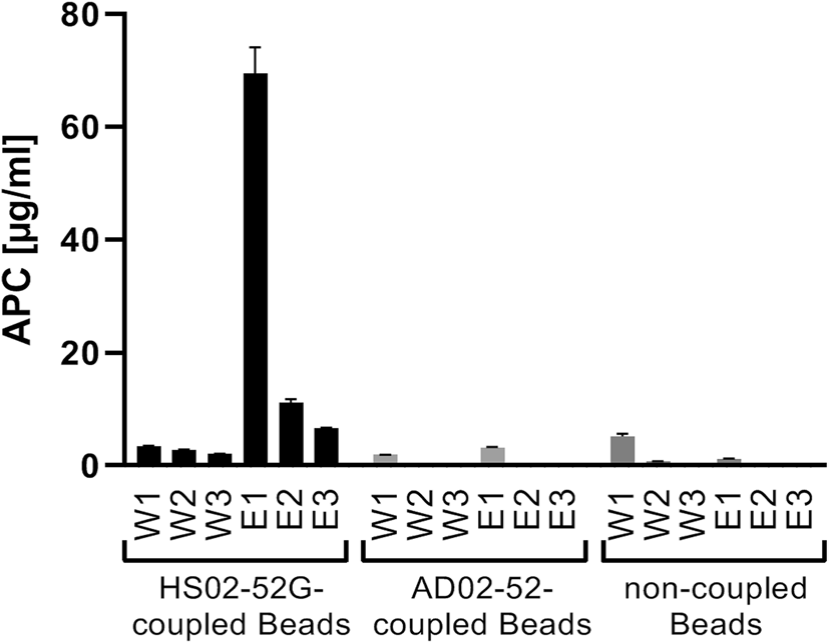
Specificity of aptamer-loaded beads. Capturing and elution results of APC using MyOne beads coupled to APC-specific HS02-52G aptamer, negative control sequence AD02-52, or non-coupled MyOne beads. The amount of APC which was captured from binding buffer containing 10 µg APC in 500 µl of binding buffer (5 µg/ml) and released during consecutive steps of washing (W1 to W3) or elution (E1 to E3) was quantified by an activity assay using an APC standard curve. Data are shown as mean ± SD of three measurements.

In order to exclude the unspecific binding of APC to the beads, non-loaded SMB or SMB loaded with an antisense sequence of HS02-52G, AD02-52 were introduced to the same capturing and elution system. As shown in Fig. 4, only the application of HS02-52G-loaded beads enabled the isolation of APC from binding buffer, demonstrating the specificity of loaded aptamer for APC capturing. In addition, the release of high APC amount from non-loaded SMB in the first washing step might demonstrate higher unspecific adsorption of APC to non-loaded beads in comparison to loaded beads either with the specific or unspecific aptamer.

**Figure 5:**
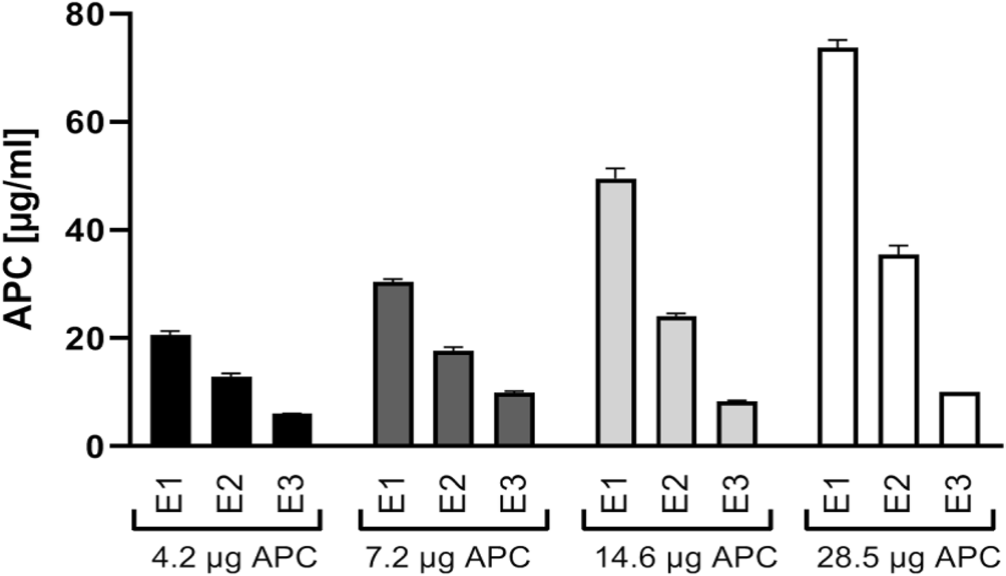
Capturing and elution of APC using HS02-52G coupled MyOne beads. Different amounts of APC were added to 500 µl binding buffer followed by APC capturing with MyOne beads coupled to HS02-52G aptamer and elution using elution buffer containing 5 mM EDTA. The eluted APC in consecutive elution steps (E1 to E3) was quantified by an activity assay using an APC standard curve. Data are shown as mean ± SD of three measurements.

To evaluate the capturing capacity of the selected MyOne beads, increasing amounts of APC were spiked to 500 µl of binding buffer and the amount of APC released during consecutive washing steps or eluted in elution steps was monitored. Data showed that the loss of APC observed during washing steps increased with the amount of APC added to the starting solution. This might be explained by the overload of loosely-bound APC molecules on the beads (Table 1, Fig. 5) or by trapping the binding buffer which contained a high concentration of non-bound APC, within the free spaces among the beads. The amount of captured APC increased with the initial amount of APC introduced to the beads. However, the yield of capturing in relation to the initial APC amount decreased drastically, showing ineffectiveness of subjecting high initial APC concentrations to capturing procedure.

**Table 1:** Characterization of capturing capacity of HS02-52G-coupled MyOne beads using increasing amounts of APC in binding buffer.

Starting APC amount, µg (pmol)	Captured APC, µg (pmol)	Captured APC compared to the starting amount of APC (%)
4.2 (74.7)	1.9 (33.8)	45
7.2 (128.1)	2.8 (49.8)	39
14.6 (259.8)	4.1 (72.9)	28
28.5 (507.1)	5.95 (105.9)	21

**Figure 6:**
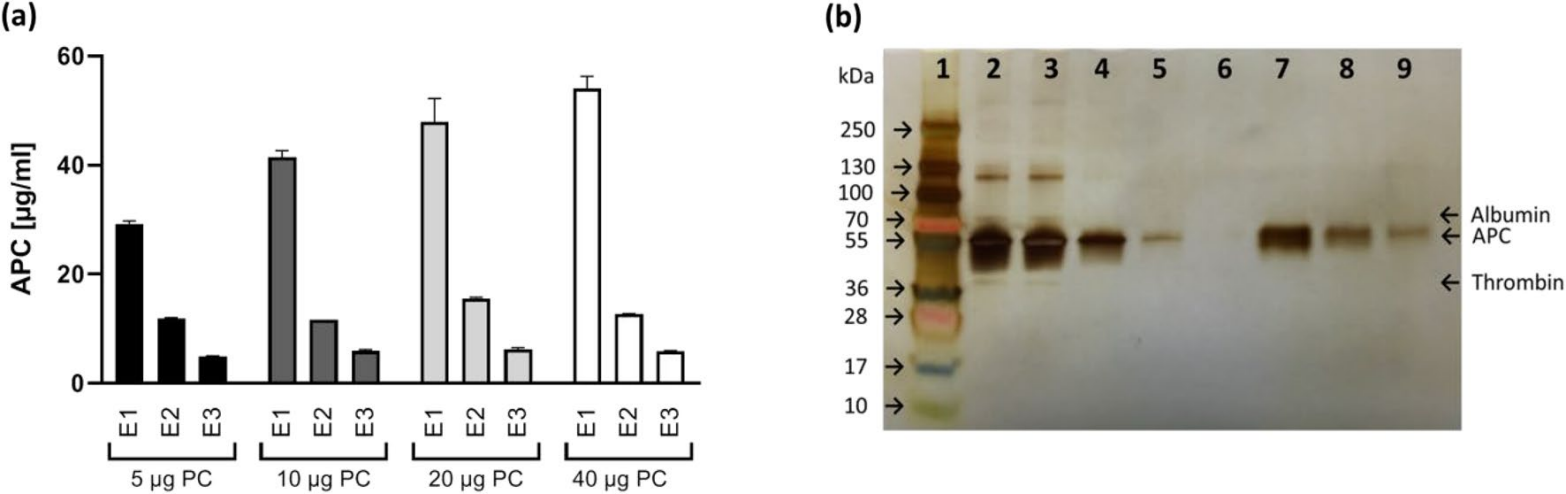
Evaluation of capture and elution process using PC. (**a**) APC generated by activation of different PC amounts was captured using HS02-52G coupled MyOne beads and eluted using elution buffer containing 5 mM EDTA. The APC content in each elution (E1 to E3) step was quantified using the APC activity assay and an APC standard curve. Data are shown as mean ± SD of three measurements. (**b**) The purity of the captured APC was analyzed qualitatively on SDS-PAGE gel. Samples from 10 µg PC starting solution before and after capturing, and corresponding samples of washing and elution fractions were loaded on SDS-PAGE gel and subjected to silver-staining. Lane 1, protein ladder; lane 2, supernatant before capturing; lane 3, supernatant after capturing; lane 4, first washing fraction; lane 5, second washing fraction; lane 6, third washing fraction; lane 7, first elution fraction; lane 8, second elution fraction; lane 9, third elution fraction.

Finally, to confirm that the MyOne beads loaded with HS02-52G are able not only to capture APC from a purified system containing only APC, but also from the activation mixture containing generated APC, residual amounts of PC as well as human thrombin and hirudin, different amount of PC (5 µg to 40 µg Ceprotin) were subjected first to activation process (see section PC activation) and then to the optimized capture and elution process. The obtained data showed that the generated APC was captured easily from the activation mixture. Increasing the initial amount of PC from 5 to 40 µg was associated with a slight increase of the captured amount from 4.6 to 7.3 µg, however higher loss of APC during washing steps was observed as well. Increasing the initial amount of PC to more than 20 µg did not increase more the captured yield indicating that the maximum loading capacity of the beads was reached (Fig. 6a). The high purity of captured APC from the activation mixture which contained 10 µg PC was confirmed by loading the activation mixture, the same as products of washing and elution steps, on SDS-PAGE gel followed by silver staining (Fig. 6b, Figure S5 and S6). According to the fact that APC and PC have close molecular weights and discrimination of their corresponding bands on gel might be difficult, samples of the supernatant before and after capturing, the same as first elution fraction resulted from 5 µg, 10 µg, 20 µg, and 40 µg PC activation were subjected to an enzyme-linked fluorescence assay (VIDAS Protein C) which is only reactive to PC and is not interfered by APC.

The data showed in Table 2 confirmed that although PC activation yield reduced when higher PC amount was subjected to the activation process, but this increasing residual amount of PC didn’t lead to higher co-purification of PC with APC which confirms the specificity of HS02-52G aptamer in capturing APC.

**Table 2:** PC quantification in the supernatant of superparamagnetic beads loaded with HS02-52G aptamer before and after capturing process, the same as the first elution fraction.

Starting PC amount, µg	Starting PC concentration (µg/ml)	PC (µg/ml)		
		SB^1^	SA^2^	E1^3^
5	10	2.02	2.16	< 0.2^4^
10	20	3.89	3.99	< 0.2
20	40	6.38	6.3	< 0.2
40	80	12.06	12.05	< 0.2

^1^SB, supernatant before capturing ; ^2^SA, supernatant after capturing ; ^3^E1, first elution fraction ; ^4^according to the lowest dilution factor needed for elution fractions, 0.2 µg/ml is the minimum detectable PC concentration which can be detected.

## Conclusions

Oligonucleotide-based affinity purification strategies for selective extraction of a target protein from complex matrices are an emerging field. In this study, we have successfully optimized PC activation and developed and validated an aptamer-facilitated protein purification process using superparamagnetic beads. The gentle elution of aptamer-bound active enzyme using EDTA circumvents protein degradation which might occur using other invasive elution conditions. This method can be utilized for rapid PC activation and isolation of highly purified APC. The purified APC might be used directly for downstream processes in which high concentrations of pure and active APC is needed.

## Methods

### Chemicals and oligonucleotides

All basic chemicals were purchased from Merck/Sigma-Aldrich (Darmstadt, Germany) unless otherwise noted. Ceprotin was obtained from Baxter AG (Vienna, Austria). Plasma derived protein C (pPC), activated protein C (APC), and human α-thrombin were purchased from Hematologic Technologies Inc. (HTI, Essex Junction, VT, USA). PCa-5791 fluorogenic peptide substrate (Pyr-Pro-Arg-AMC) was bought from Loxo (Dossenheim, Germany). Refludan was purchased from Bayer Health Care (Leverkusen, Germany). Dynabeads MyOne Carboxylic Acid beads (MyOne) and Dynabeads M-270 Carboxylic Acid beads were purchased from Life technologies (Karlsruhe, Germany). SDS-PAGE gradient gel, 2 × Laemmli loading buffer, α-mercaptoethanol, colloidal coomassie stain G-250 and destaining solution were purchased from Bio-rad (Hercules, CA, USA), protein ladder was purchased from Affinity Biologicals (Ancaster, Canada). SilverXpress sliver staining kit was purchased from ThermoFisher scientifics (Waltham, MA, USA). Berichrom Protein C Kit was purchased from Siemens Healthcare Diagnostics (Marburg, Germany) and VIDAS Protein C test kit was bought from Biomerieux (Nürtingen, Germany).

The ssDNA oligonucleotides were ordered from Ella Biotech (Martinsried, Germany) having the corresponding sequences linked either with TEG-biotin or with HEG-Amino at the 3´end: **HS02-52G**, 5’-GCC TCC TAA CTG AGC TGT ACT CGA CTT ATC CCG GAT GGG GCT CTT AGG AGG C -3’, **NB1-83** 5´-AAG CAG TGG TAA GTA GGT TGA CAC ATT AGG GCG GGG TAC TCC TAT CAC GTA TGG GGG CCT GTG TCT CTT CGA GCA ATC CAC AC-3´,**NB1-46** 5´-GAC ACA TTA GGG CGG GGT ACT CCT ATC ACG TAT GGG GGC CTG TGT C-3´, **NB2-81** 5´-GAT TGT TAC TGT CAC GAG GAT ATC ACG TAT GGG GGG CCG GCA TGA GGG CCG CGC GTG ACA ATA GCA CAT TAG TTC AGA TAC-3´, **NB2-57G** 5´-TAC TGT CAC GAG GAT ATC ACG TAT GGG GGG CCG GCA TGA GGG CCG CGC GTG ACA GTA-3´ and **AD02-52**, 5´-GCC TCC TAA GAG CCC CAT CCG GGA TAA GTC GAG TAC AGC TCA GTT AGG A GG C -3´^18^.

### Formulation of used buffers

PC-activation buffer: 20 mM Tris–HCl, 100 mM NaCl, 1 mM EDTA, pH 7.4; SDS-PAGE running buffer: 25 mM Tris–HCl, 192 mM glycine, 0.1% SDS, pH 8.3; binding buffer: 10 mM Tris–HCl, 154 mM NaCl, 1 mM CaCl_2_, 0.01% BSA, pH 7.4; dissociation buffer 1: 10 mM Tris–HCl, 1 mM MgCl_2_, 1 mM CaCl_2_, 0.1% BSA, 1 M NaCl pH 7.4; dissociation buffer 2: 10 mM Tris–HCl, 0.1% BSA, 5 mM EDTA pH 7.4; washing buffer: 10 mM Tris–HCl, 154 mM NaCl, 1 mM CaCl_2_, 0.05% Tween 20, pH 7.4; elution buffer: 10 mM Tris–HCl, 154 mM NaCl, 5 mM EDTA, pH 7.4; MES buffer: 100 mM MES, pH 4.8; substrate buffer: 10 mM Tris–HCl, 154 mM NaCl, 4 mM CaCl_2_, pH 8.5; Tris-Tween20 (TT): 250 mM Tris–HCl, 0.01% Tween 20, pH 8; Tris–EDTA (TE): 10 mM Tris–HCl, 1 mM EDTA, 0.02% sodium azide, pH 8.

### Protein C activation

Ceprotin (Baxter AG) lyophilized powder containing 500 IU of PC was dissolved in 2.5 mL water for injection to reach 250 IU/ml concentration. Berichrom Protein C Kit (Siemens Healthcare Diagnostics) was used to determine the PC concentration in comparison to PC standard solutions prepared from commercially available plasma-derived PC (pPC, HTI). To achieve the optimum generation of APC, as the first step, thrombin concentration was optimized. Briefly, Ceprotin (62.5 μg/ml equal to 1 µM) was incubated with different concentrations of human α-thrombin (0.78 µg/ml to 12.5 µg/ml) in activation buffer. After 1 h of incubation, samples were subjected to the APC activity assay (see the APC activity assay section). To avoid the unspecific cleavage of APC-specific substrate by thrombin, residual activity of thrombin was inhibited by addition of recombinant hirudin (Refludan, Bayer Health Care). In order to identify the optimum hirudin concentration, 2.5, 12.5, and 20 µg/ml thrombin was incubated with increasing concentrations of hirudin (14 nM to 14 µM) in activation buffer. After incubation for 30 min, 50 µl of the mixture were transferred to the wells of a black F16 Fluoronunc module (ThermoFisher Scientific, Nunc) containing 50 µl of 10 mM fluorogenic thrombin substrate (Boc-Asp(OBzl)-Pro-Arg-AMC) and thrombin catalyzed substrate hydrolysis was monitored at λ_ex_ of 360 nm and λ_em_ of 460 nm using a Synergy 2 microplate reader (BioTek, Bad Friedrichshall, Germany).

The optimum incubation time of PC and thrombin was determined by incubation of 62.5 μg/ml PC with 3.12 µg/ml thrombin for up to 24 h and the APC activity was monitored using the APC activity assay described below.

In order to subject the generated APC to further downstream experiments, a buffer exchange step was included. Briefly the activation mixture passed through the centrifugal filter units (Amicon Ultra-2 Ultracell—NMWL 10 kDa, Merck, Darmstadt, Germany) and the activation buffer was exchanged with binding buffer.

### APC activity assay and PC quantification assay

The activity of APC was assessed using an APC-specific fluorogenic peptide substrate (PCa-5791). Briefly, 50 µl of a 1:50 dilution of the sample in assay buffer were transferred to the wells of white F8 FluoroNunc modules [Thermo Fisher Scientific (Nunc)]. Subsequently, 50 µl of 300 µM PCa-5791 were added and substrate hydrolysis rates were recorded at λ_ex_ of 360 nm and λ_em_ of 460 nm using the Synergy 2 microplate reader. The same activity assay was used to determine the stability of APC in binding buffer. A stock solution containing 160 nM APC was kept on ice and aliquots were removed and incubated at 37 °C at selected time points. At the end of the incubation time 50 µl of a 1:10 dilution of each sample were added to the wells of white F8 FluoroNunc modules followed by addition of 50 µl of the 300 µM PCa-5791. The APC activity was compared to the activity of the APC stock solution which was kept for the maximum incubation time on ice and presented as the activity in percentage.

PC quantification was performed in an enzyme-linked fluorescent assay (ELFA) using VIDAS Protein C assay. Briefly, samples were recalcified with CaCl_2_ and diluted 1:3 to 1:10 in D-PBS buffer containing 1 mM MgCl_2_ and 1 mM CaCl_2_. Then diluted samples were incubated in the SPR cuvettes of the system which were coated with PC-specific antibody. Further, captured PC was quantified after subsequent addition of a secondary antibody linked to alkaline phosphatase and 4-methyl umbelliferyl phosphate (4-MUP) substrate.

### Biolayer interferometry analysis

Biolayer Interferometry (BLI) technology was applied^40^ first to determine the appropriate aptamer to be loaded on beads and then to evaluate the dissociation kinetics of APC from aptamer molecules loaded on a High-Precision Streptavidin (SAX) Biosensor (Pall Life Sciences) when the biosensor immersed in different dissociation buffers (dissociation buffer 1–3). A BLItz system (Pall Life Sciences, Dreieich, Germany) and the Blitz 1.2.1.5 software package were used for corresponding analysis.

To identify the best aptamer for capturing, the SAX biosensor was hydrated for 10 min followed by loading of 3´-biotinylated HS02-52G, NB1-83, NB1-46, NB2-81, and NB2-57G aptamers (using a 500 nM solution of the aptamer in binding buffer) for 2 min. The loaded biosensor was equilibrated for 30 s in binding buffer followed by immersing in the same buffer containing increasing concentrations of APC to calculate the association rate constant (ka [kon]). Thereafter, for determination of dissociation rate constants (kd [koff]), the biosensor was lowered into a tube containing 500 µL of the same binding buffer. The K_D_ was calculated out of ka and kd of using different concentrations of APC.

To determine the optimum dissociation buffer, the SAX biosensor was loaded with the aptamer and was used for APC capturing out of binding buffer containing 500 nM APC. Then, the biosensor was lowered into a tube containing 500 µL of either binding buffer or each dissociation buffer 1 or 2 and the dissociation kinetic was studied. All measurements were performed at a shaking speed of 2,200 rpm.

### Aptamer loading on carboxylic acid magnetic beads

Five milligrams of Dynabeads MyOne carboxylic acid beads (MyOne) or 15 mg of Dynabeads M-270 carboxylic acid beads (M-270) were washed using MES buffer followed by resuspension of the beads in 300 µl of the same buffer. HS02-52G aptamer (25 nmol) or AD02-52 having HEG-Amino at the 3´end was diluted in 150 µl MES buffer and mixed with 150 µl 1 M EDC in MES buffer. The aptamer-EDC mixture was subsequently added to the magnetic beads and mixed by vortexing for 10 s. The first sub-sampling was performed immediately after mixing, and then the mixture was mixed thoroughly overnight followed by the second sub-sampling. The loaded beads were washed 3 × using Tris-Tween20 (TT) buffer and resuspended in 500 µl Tris–EDTA (TE) buffer. The loading yield was calculated out of sub-samples at the beginning and end point of the loading process.

### Assessment of loading of aptamers on superparamagnetic beads

The SMBs which were coupled to either HS02-52G aptamer or non-binder AD02-52 aptamer, the same as non-loaded MyOne beads were diluted 1:100 in binding buffer and incubated for 30 min with SYBR Green (1:10,000 dilution) under rigorous shaking. Then the beads were washed and diluted 1:50 in binding buffer. Subsequently, the samples were transferred to 12 × 75 mm polypropylene tubes (Beckman Coulter) and subjected to a Navios EX flow cytometer (Beckman Coulter, Krefeld, Germany) for assay readout. For each reaction, 10,000 bead events were recorded (FSC/SSC or FL1) and associated binding of SYBR Green to the beads was identified by corresponding FCS/SSC- and positive events at FL1.

### Assessment of binding and release of captured molecules to/from loaded magnetic beads

To evaluate the capturing capacity of loaded beads, different amounts of APC (2.5 µg, 5 µg, 10 µg and 20 µg) were dissolved in 500 µl binding buffer and incubated with loaded beads under a shaking speed of 1,100 rpm at RT. After 30 min of incubation, beads were washed 3 × using washing buffer. Captured APC was eluted from the beads by incubation of beads with 50 µl elution buffer for three times, each 5 min. The starting solution, supernatant of the beads after capturing and, three washing and elution fractions were subjected to the APC activity assay. In order to evaluate the unspecific binding of APC to non-loaded beads as well as beads loaded with non-specific oligonucleotides, the capturing and releasing of 10 µg APC in binding buffer to/from beads in both conditions were tested. The optimum adjustment was applied for capturing of generated APC out of the activation mixture using Ceprotin.

### Purity evaluation on SDS-PAGE gel

Yield and quality of captured APC either from binding buffer or from activation mixture were tested on gradient SDS-PAGE gel followed by silver staining.

Briefly, sample containing approximately 2 µg protein in Tris–HCl buffer was mixed with the same volume of 2 × Laemmli loading buffer containing 5% α-mercaptoethanol and heated for 8 min followed by cooling down on ice. Subsequently, samples were loaded on a gradient SDS-PAGE gel (Bio-rad) and subjected to electrophoresis. The bands were visualized by silver staining following the manufacturer’s instructions.

## Supplementary Information

Supplementary Information.

## Data Availability

The data that supports the generation of oligonucleotides used in this study are openly available in Zenodo.org at https://zenodo.org/record/5878873#.Yege9lVKiJA. The molecular evolutionary relations, ligand tracing and tracking of defined sequences through successive selection rounds were performed using COMPAS software (AptalT GbmH, Planneg, Germany)^41,42^. The motif similarity and finding of consensus sequence was done using multiple sequence alignment program Clustal Omega from EMBL-EBI (https://www.ebi.ac.uk/Tools/msa/clustalo/; December 2016)^43^.

## References

[1] Griffin JH, Fernandez JA, Gale AJ, Mosnier LO (2007). Activated protein C. J. Thromb. Haemost..

[2] Gruber A, Griffin JH (1992). Direct detection of activated protein C in blood from human subjects. Blood.

[3] Okajima K (1990). Effect of protein C and activated protein C on coagulation and fibrinolysis in normal human subjects. Thromb. Haemost..

[4] Griffin JH, Zlokovic BV, Mosnier LO (2012). Protein C anticoagulant and cytoprotective pathways. Int. J. Hematol..

[5] Berg JM, Tymoczko JL, Stryer L (2002). The purification of proteins is an essential first step in understanding their function. Biochemistry.

[6] Tan SC, Yiap BC (2009). DNA, RNA, and protein extraction: The past and the present. J. Biomed. Biotechnol..

[7] Boxi A, Parikh I, Radhika BS, Shryli KS (2020). Current trends in protein purification: A review. Int. J. Sci. Res. Sci. Technol..

[8] Scopes RK (1995). Overview of protein purification and characterization. Curr. Protoc. Protein Sci..

[9] Labrou NE (2014). Protein purification: An overview. Protein Downstream Processing.

[10] Kisiel W, Davie EW, Protein C (1981). [26] Protein C. Methods in Enzymology : Proteolytic Enzymes.

[11] Esmon CT, Esmon NL, Le Bonniec BF, Johnson AE (1993). Protein C activation. Methods in Enzymology: Proteolytic Enzymes in Coagulation, Fibrinolysis, and Complement Activation Part A: Mammalian Blood Coagulation Factors and Inhibitors.

[12] Dong W (2015). Activated Protein C ameliorates renal ischemia-reperfusion injury by restricting Y-box binding protein-1 ubiquitination. J. Am. Soc. Nephrol..

[13] Ranjan S (2017). Activated protein C protects from GvHD via PAR2/PAR3 signalling in regulatory T-cells. Nat. Commu..

[14] Isermann B (2007). Activated protein C protects against diabetic nephropathy by inhibiting endothelial and podocyte apoptosis. Nat. Med..

[15] Tuerk C, Gold L (1990). Systematic evolution of ligands by exponential enrichment: RNA ligands to bacteriophage T4 DNA polymerase. Science.

[16] Ellington AD, Szostak JW (1990). In vitro selection of RNA molecules that bind specific ligands. Nature.

[17] Hamedani NS (2021). Functional and Structural characterization of nucleic acid ligands that bind to activated coagulation factor XIII. J. Clin. Med..

[18] Hamedani NS (2020). Selective modulation of the protease activated protein C using exosite inhibiting aptamers. Nucl. Acid Therap..

[19] Forier C (2017). DNA aptamer affinity ligands for highly selective purification of human plasma-related proteins from multiple sources. J. Chromatogr. A.

[20] Walter J-G, Stahl F, Acheper T (2012). Aptmaer as affinity ligands for downstream processing. Eng. Life Sci..

[21] Liu H (2017). An oligosorbent-based aptamer affinity column for selective extraction of aflatoxin B(2) prior to HPLC with fluorometric detection. Mikrochimica Acta.

[22] Chapuis-Hugon F, Du Boisbaudry A, Madru B, Pichon V (2011). New extraction sorbent based on aptamers for the determination of ochratoxin A in red wine. Anal. Bioanal. Chem..

[23] Safarik I, Safarikova M (2004). Magnetic techniques for the isolation and purification of proteins and peptides. Biomagn. Res. Technol..

[24] Lönne M (2015). Development of an aptamer-based affinity purification method for vascular endothelial growth factor. Biotechnol. Rep..

[25] Çimen D, Bereli N, Denizli A (2020). Metal-chelated magnetic nanoparticles for protein C purification. Separat. Sci. Technol..

[26] Muller J (2009). An exosite-specific ssDNA aptamer inhibits the anticoagulant functions of activated protein C and enhances inhibition by protein C inhibitor. Chem. Boil..

[27] Esmon CT (2003). The protein C pathway. Chest.

[28] Ye J, Esmon NL, Esmon CT, Johnson AE (1991). The active site of thrombin is altered upon binding to thrombomodulin. Two distinct structural changes are detected by fluorescence, but only one correlates with protein C activation. J. Boil. Chem..

[29] Rezaie AR, Yang L (2003). Thrombomodulin allosterically modulates the activity of the anticoagulant thrombin. Proc. Natl. Acad. Sci. U.S.A.

[30] Yang L, Manithody C, Rezaie AR (2006). Activation of protein C by the thrombin-thrombomodulin complex: Cooperative roles of Arg-35 of thrombin and Arg-67 of protein C. Proc. Natl. Acad. Sci. U.S.A.

[31] Fenton JW, Villanueva GB, Ofosu FA, Maraganore JM (1991). Thrombin inhibition by hirudin: How hirudin inhibits thrombin. Haemostasis.

[32] Cai S (2018). Investigations on the interface of nucleic acid aptamers and binding targets. The Analyst.

[33] Hianik T, Ostatná V, Sonlajtnerova M, Grman I (2007). Influence of ionic strength, pH and aptamer configuration for binding affinity to thrombin. Bioelectrochemistry.

[34] Zhu G, Walter J-G (2015). Aptamer-modified magnetic beads in affinity separation of proteins. Methods in Molecular Biology.

[35] Hamedani NS (2015). Capture and Release (CaR): a simplified procedure for one-tube isolation and concentration of single-stranded DNA during SELEX. Chem. Commun..

[36] Hamedani NS (2016). In vitro evaluation of aptamer-based reversible inhibition of anticoagulant activated protein C as a novel supportive hemostatic approach. Nucl. Acid Ther..

[37] Walter J-G, Kökpinar O, Friehs K, Stahl F, Scheper T (2008). Systematic investigation of optimal aptamer immobilization for protein-microarray applications. Anal. Chem..

[38] Lim HK, Kim IH, Nam HY, Shin S, Hah SS (2013). Aptamer-based alternatives to the conventional immobilized metal affinity chromatography for purification of his-tagged proteins. Anal. Lett..

[39] Perret G, Boschetti E (2018). Aptamer affinity ligands in protein chromatography. Biochimie.

[40] Concepcion J (2009). Label-free detection of biomolecular interactions using BioLayer interferometry for kinetic characterization. Combinat. Chem. High Through. Screen..

[41] Pfeiffer F (2018). Systematic evaluation of error rates and causes in short samples in next-generation sequencing. Sci. Rep..

[42] Blank M, Mayer G (2016). Next-generation analysis of deep sequencing data: Bringing light into the black box of SELEX experiments. Nucleic Acid Aptamers: Selection, Characterization, and Application.

[43] Goujon M (2010). A new bioinformatics analysis tools framework at EMBL-EBI. Nucl. Acids Res..

