# Supplementary material for: Aptamer loaded superparamagnetic beads for selective capturing and gentle release of activated protein C
Source: Sci Rep. 2022 Apr 30;12:7091. doi: 10.1038/s41598-022-11198-5 (PMC9056527; doi:10.1038/s41598-022-11198-5)
Supplement: Supplementary file 1 — Supplementary Information. [file 41598_2022_11198_MOESM1_ESM.docx]

**Electronic Supplementary Information**

**Aptamer loaded superparamagnetic beads for selective capturing and gentle release of activated protein C**

Nasim Shahidi Hamedani^1#*^, Felix Lucian Happich^1#^, Eva-Maria Klein^1^, Heiko Rühl^1^, Günter Mayer^2^, Johannes Oldenburg^1^, Jens Müller^1^, Bernd Pötzsch^1^

^1^ Institute of Experimental Hematology and Transfusion Medicine, University Hospital Bonn, Bonn, Germany

^2^ Life and Medical Sciences Institute, University of Bonn, Bonn, Germany

* Corresponding author: Nasim.shahidi_hamedani@ukbonn.de

# these authors contributed equally to this work


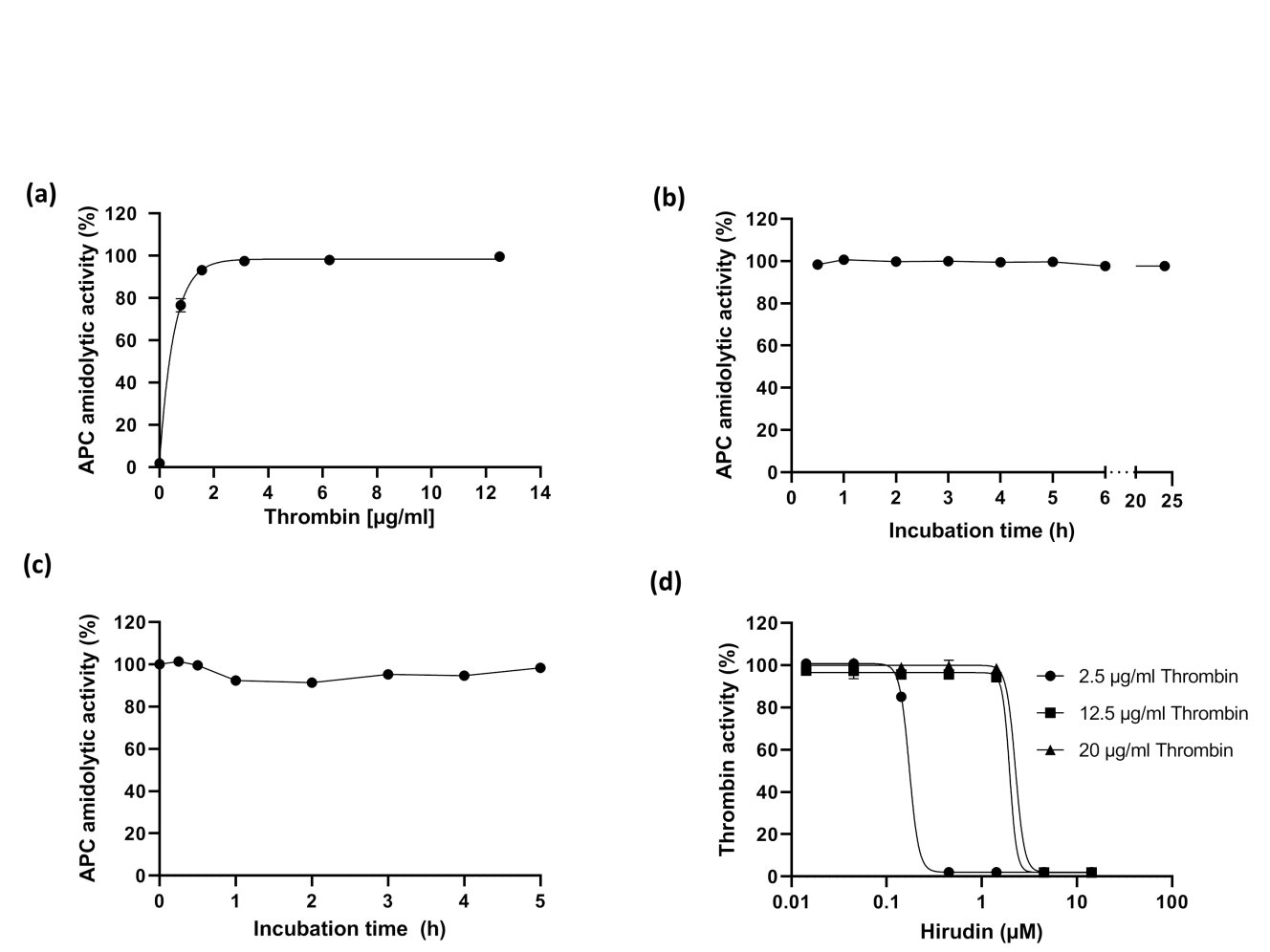


**Figure S1**. Optimization of PC activation. (a) Optimization of thrombin concentration. PC (62.5 μg/ml) was incubated with different concentrations of thrombin. After 1 h of incubation, hirudin was added to the mixture and APC activity was measured by mixing of 50 µl of a 1:50 dilution of the samples and 50 µl of PCa-5791 peptide substrate. (b) Incubation time optimization. PC (62.5 μg/ml) was mixed with 3.12 µg/ml thrombin. After the indicated incubation times, samples were diluted 1:50 and subjected to the APC activity assay. (c) APC stability in activation buffer. APC (160 nM in activation buffer) was incubated at 37°C for the indicated intervals followed by 1:10 dilution of the samples and activity measurement (d) Optimization of hirudin concentration. Different concentrations of thrombin were incubated with increasing concentrations of hirudin (14 nM to 14 µM) in activation buffer. After incubation for 30 min, 50 µl of the mixture were transferred to the wells of a black F16 Fluoronunc module (Thermo Fisher Scientific, Nunc) containing 50 µl of 10 mM fluorogenic thrombin substrate (Boc-Asp(OBzl)-Pro-Arg-AMC) and thrombin catalysed substrate hydrolysis was monitored using a Synergy 2 microplate reader.


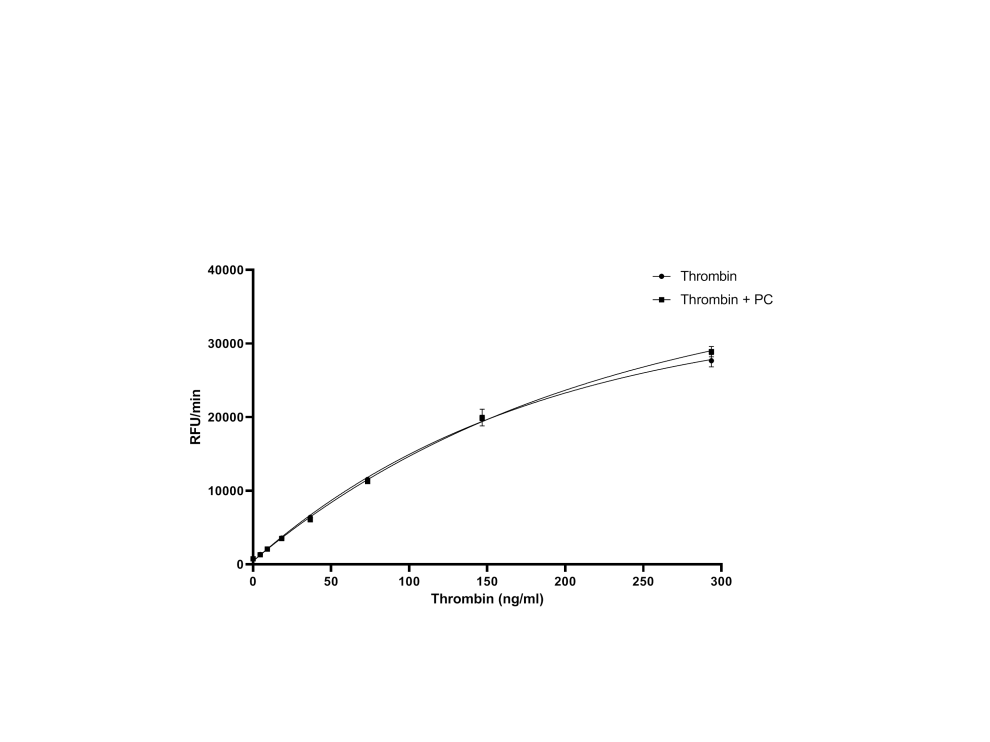


**Figure S2.** Monitoring the specificity of the PCa-5791 fluorogenic peptide substrate. Fifty microliters of of thrombin at indicated final concentrations which was pre-incubated with or without PC (5 µg/ml) were mixed with 50 µl of 600 µM PCa-5791 and the hydrolysis rate of the substrate was monitored at λ_ex_ 360 nm and λ_em_ of 460 nm using a microplate reader. Data are presented as mean ± s.d. of duplicate measurements.


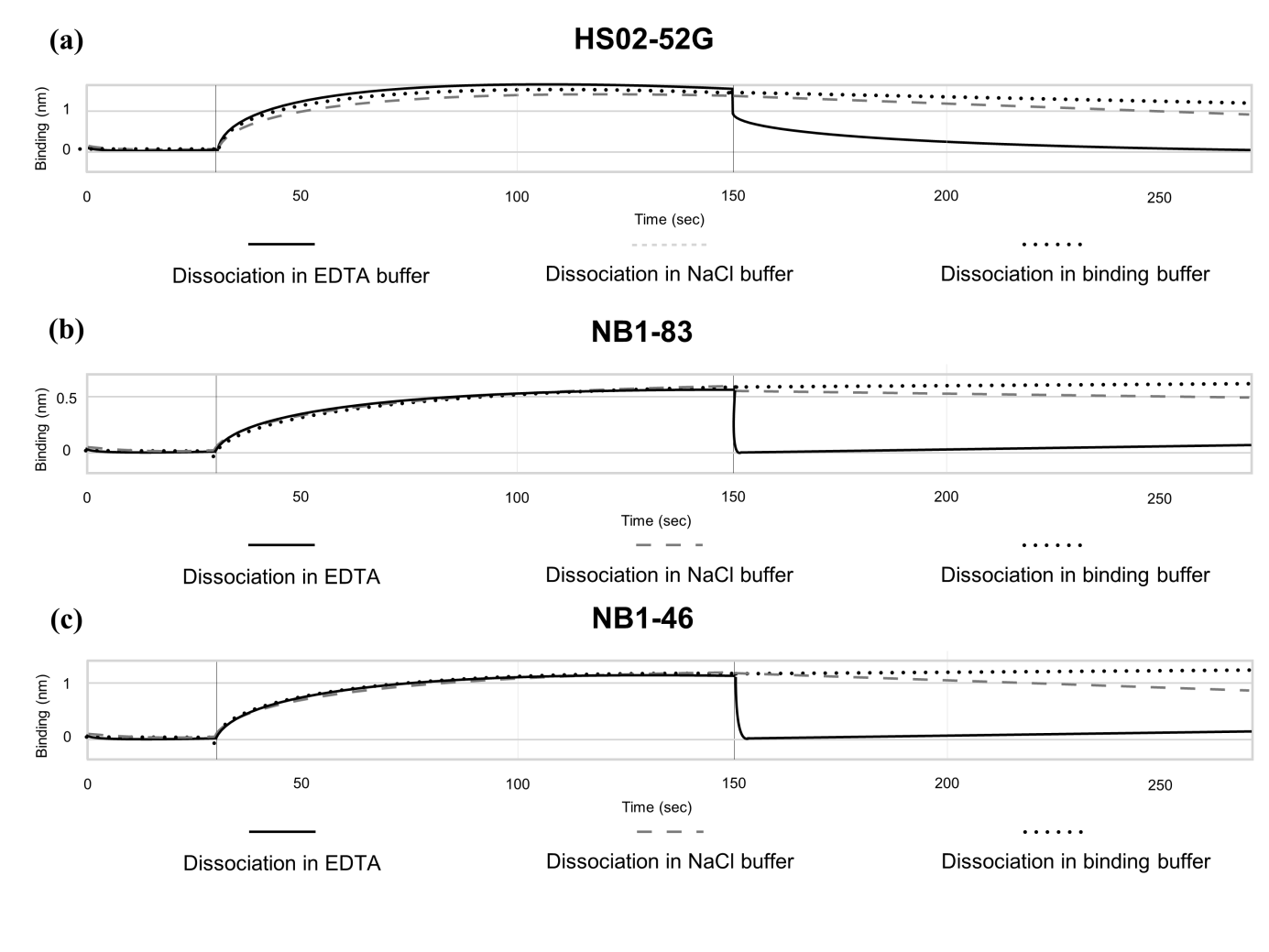

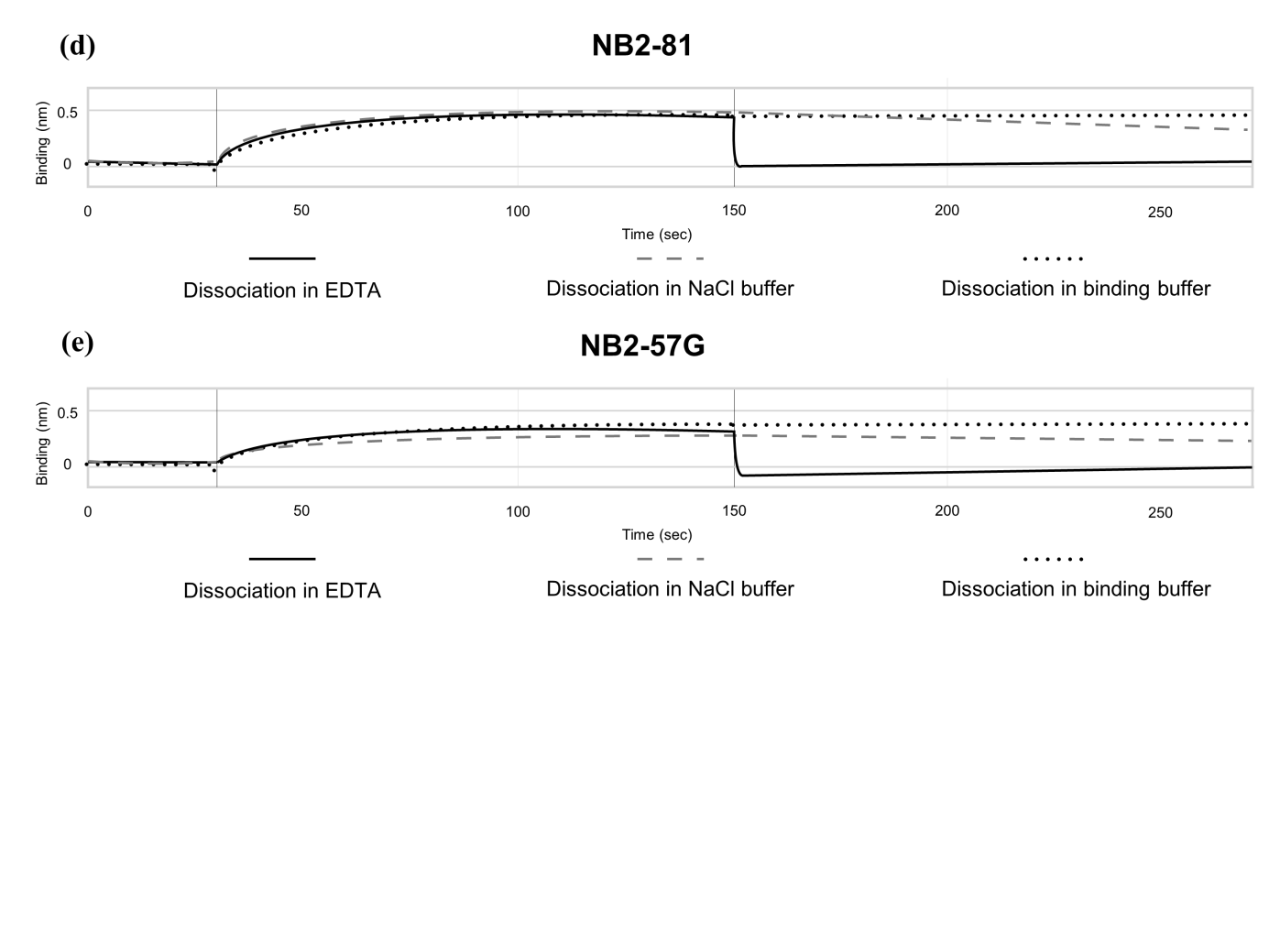


**Figure S3.** Dissociation of APC from immobilized aptamers using biolayer interferometry (BLI). Biotinylated aptamers (a) HS02-52G, (b) NB1-83, (c) NB1-46, (d) NB2-81, (e) NB2-57G were immobilized on the surface of streptavidin-coated biosensors by immersing of the hydrated biosensor in binding buffer containing 500 nM of each aptamer followed by association of 500 nM APC. The APC dissociation was performed in binding buffer either without or with 5 mM EDTA or 1 M NaCl.


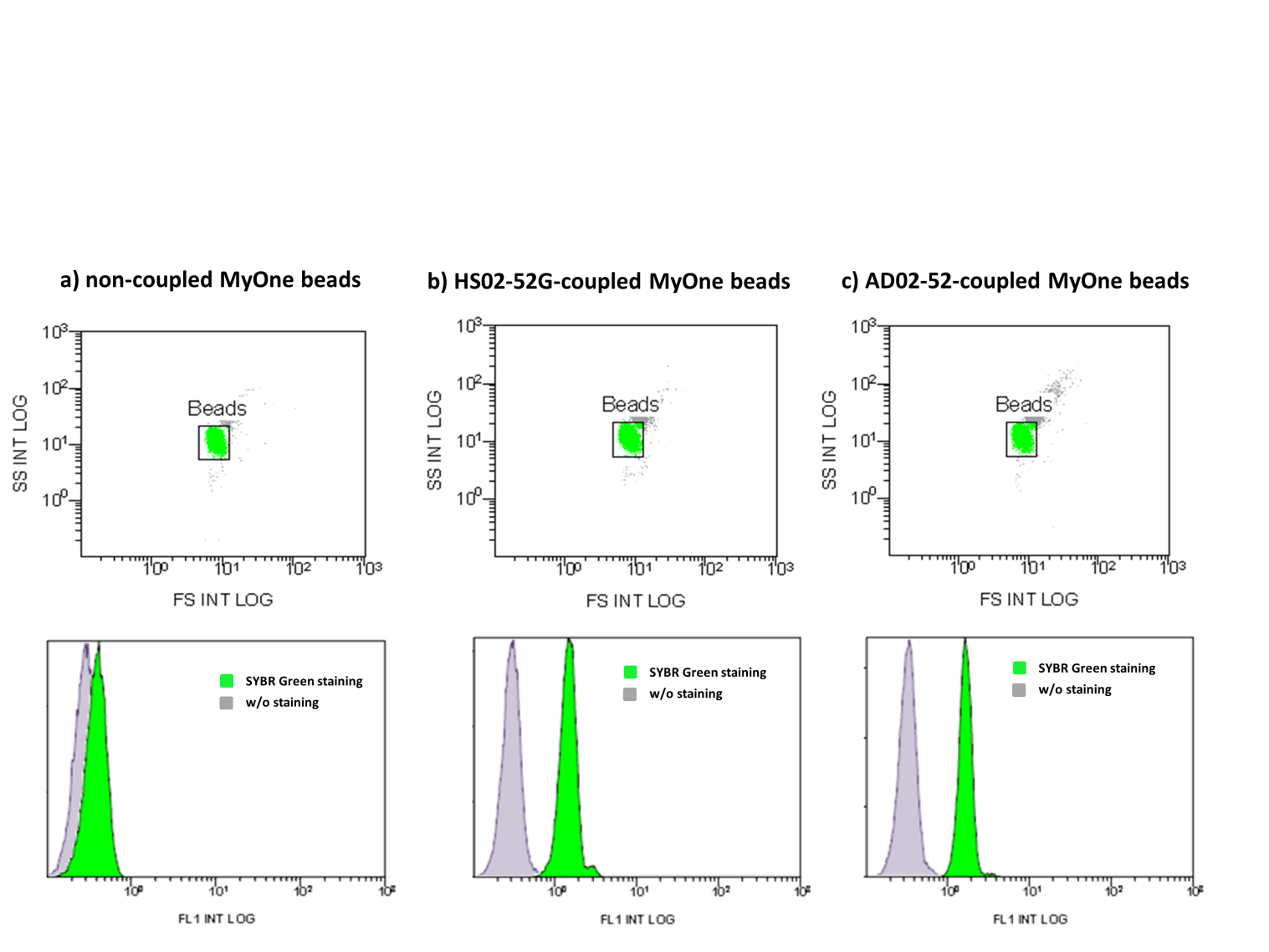


**Figure S4.** MyOne superparamagnetic beads which were not coupled to aptamer molecules (a) or coupled to either HS02-52G (b) or AD02-52 (c) were incubated with (green) or without (gray) the nucleic acid stain SYBR Green (1:10,000 dilution). After incubation, samples are diluted and beads were gated by FSC/SSC- (upper panels) or fluorescence (SYBR Green, FL1, lower panel) emission characteristics.


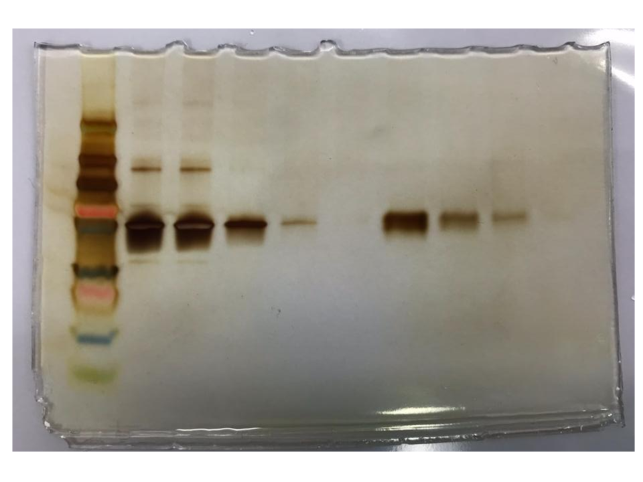


**Figure S5.** The original SDS-PAGE gel image corresponds to activation of 10 µg PC in 500 µl binding buffer before and after capturing, washing and elution fractions. From Left to the right, Molecular weight ladder, supernatant before capturing, supernatant after capturing, washing fraction 1, washing fraction 2, washing fraction 3, elution fraction 1, elution fraction 2, elution fraction 3.


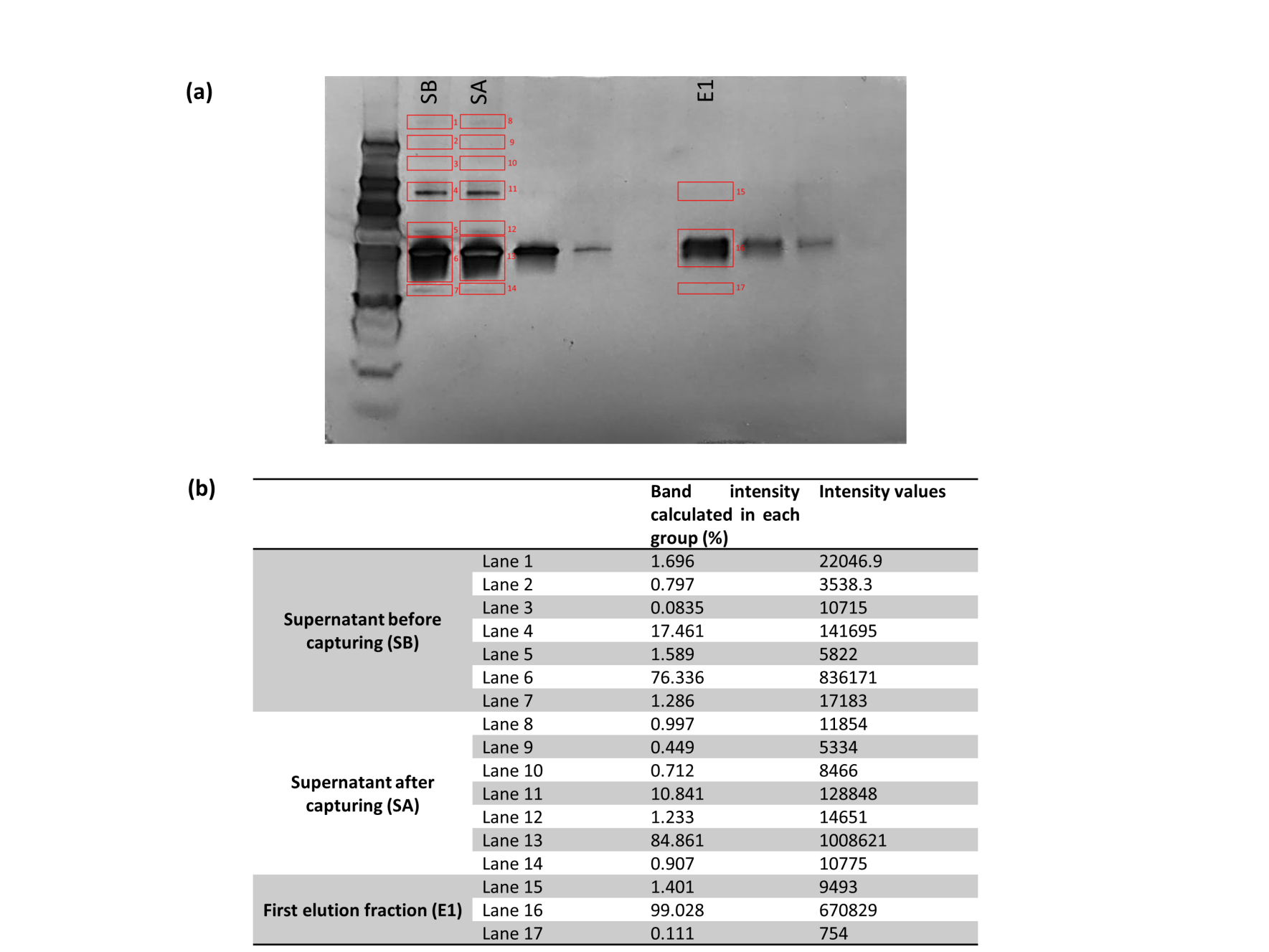


**Figure S6.** (a) Band intensity quantification on SDS-PAGE gel corresponds to samples resulted from activation of 10 µg PC starting solution before and after capturing, washing, and elution fractions. Band intensity quantification was performed using GelQuantNET software on 08.03.2022 available at <http://biochemlabsolutions.com/GelQuantNET.html>. (b) Band intensity values and percentrage resulted from quantification of supernatant before and after APC capturing and first elution fraction. SB, supernatant before capturing; SA, supernatant after capturing, E1, first elution fraction.

**Table S1.** The association (ka) and dissociation (kd) constants calculated from binding of APC to streptavidin-coated biosensors loaded with each biotinylated aptamers and corresponding dissociation in binding buffer, elution buffer containing either 5 mM EDTA or 1M NaCl calculated by local analysis of binding curves.

| Aptamer | Dissociation solution | Ka (1/Ms) | Kd (1/s) |
| --- | --- | --- | --- |
| HS02-52G | EDTA | 6.4e4 | 2.3e-2 |
|  | NaCl | 9.5e4 | 6.7e-3 |
|  | Binding buffer | 1.2e5 | 2.5e-3 |
| NB1-83 | EDTA | 6.03e4 | n.d.* |
|  | NaCl | 6.04e4 | 1.93e-3 |
|  | Binding buffer | 5.67e4 | < e-7 |
| NB1-46 | EDTA | 9.07e3 | 7.8e-3 |
|  | NaCl | 2.29e4 | 2.4e-3 |
|  | Binding buffer | 3.88e4 | < e-7 |
| NB2-81 | EDTA | 4.1e4 | n.d. |
|  | NaCl | 3.26e4 | 1.25e-3 |
|  | Binding buffer | 1.77e4 | < e-7 |
| NB2-57G | EDTA | 2.67e4 | n.d. |
|  | NaCl | 5.66e3 | 2.93e-3 |
|  | Binding buffer | 1.84e4 | < e-7 |

* n.d. Not detected. Fast dissociation of APC from the aptamer-loaded biosensor leads to miscalculation of kd by the software.

**Table S2.** Characterization of two different types of magnetic beads modified with HS02-52G aptamer

|  | Amount of beads, mg | Total served surface, cm^2^ | Coupled aptamer, µg/mg beads (nmol/mg beads) | Aptamer coupled to the beads (% of initial amount)* |
| --- | --- | --- | --- | --- |
| MyOne  Carboxylic Acid beads | 5 | 142 | 13.3 (0.82) | 16.4 |
| M-270 Carboxylic Acid beads | 15 | 246 | 5.38 (0.22) | 13.4 |

* The initial amount of aptamer used for coupling reaction was 25 nmol.
